# The association between cognition and gait disturbance in central nervous system demyelinating disorder with mild disability

**DOI:** 10.1186/s12883-023-03210-w

**Published:** 2023-04-29

**Authors:** Min Cheol Chang, Byung Joo Lee, Dongseok Yang, Chung Reen Kim, Donghwi Park, Sunyoung Kim

**Affiliations:** 1 Department of Physical Medicine and Rehabilitation, College of Medicine, Yeungnam University, Daegu, Republic of Korea; 2 Department of Physical Medicine and Rehabilitation, Daegu Fatima Hospital, Ayang-Ro 99 Gil, Dong-Gu, Daegu, 41199 Republic of Korea; 3 Department of Physical Medicine and Rehabilitation, Ulsan University Hospital, Ulsan University College of Medicine, Ulsan, Korea; 4 Department of Neurology, Ulsan University Hospital, Ulsan University College of Medicine, 877 Bangeojin sunhwando-ro, Dong-gu, 44033 Ulsan, Korea

**Keywords:** Gait, Gait analysis, Cognitive function, Multiple sclerosis, Neuromyelitis optica

## Abstract

**Introduction:**

Gait disturbance in central nervous system (CNS) demyelinating disorders, including multiple sclerosis (MS) and neuromyelitis optica (NMO) is one of the most troublesome problems that has a direct impact on the quality of life. However, the associations between gait disturbance and other clinical variables of these two diseases have not been fully elucidated.

**Objective:**

This study aimed to evaluate gait disturbance using a computerized gait analysis system and its association with various clinical variables in patients with MS and NMO.

**Methods:**

A total of 33 patients (14 with MS and 19 with NMO) with minor disabilities, who were able to walk independently and had passed their acute phase, were enrolled in the study. Gait analysis were performed using a computer-based instrumented walkway system. (Walk-way MG-1000, Anima, Japan) Clinical variables, such as disease duration, medication, body mass index (BMI), hand grip power, and muscle mass were recorded. The Montreal Cognitive Assessment (MOCA), Beck Depression Inventory score-II (BDI), and fatigue scale were measured using the Functional Assessment of Chronic Illness Therapy-fatigue scale (FACIT-fatigue) scale. A trained neurologist scored the Expanded Disability Status Scale (EDSS).

**Results:**

Gait speed was the single parameter that showed a significant positive correlation with MOCA (*p* < 0.001). The stance phase time was the single parameter that showed a significant negative correlation with EDSS (*p* < 0.001). Hand grip strength showed a significant positive correlation with skeletal muscle mass as assessed by bioimpedance analysis (*p* < 0.05). The FACIT-fatigue scale score showed a significant negative correlation with the BDI (*p* < 0.001).

**Conclusion:**

In our patients with MS/NMO with mild disability, cognitive impairment was significantly correlated with gait speed, and the degree of disability was significantly correlated with stance phase time. Our findings may imply that early detection of a decrease in gait speed and an increase in stance phase time can predict the progression of cognitive impairment in patients with MS/NMO with mild disability.

## Introduction

Demyelinating diseases of the central nervous system (CNS), including multiple sclerosis (MS) and neuromyelitis optica (NMO), are caused by the attack of white blood cells and antibodies on the myeline sheath or the cells that produce and maintain it in the spinal cord or brain [1]. Damage to the myelin sheath interrupts nerve transmission, resulting in various neurological dysfunctions according to the involved part of the nervous system [1]. Deterioration of gait function is one of the most common neurological deficits in patients with CNS demyelinating diseases [2]. Gait function is important for independence in daily activities and is essential for the mobility and maintenance of general health in humans. Demyelinating disease-induced gait disturbances frequently result in falls and fall-related injuries. Therefore, gait disturbance in patients with CNS demyelinating diseases is one of the most critical factors that impairs activities of daily living, general health, and quality of life [2].

CNS demyelinating disease decreases gait speed and endurance, step length, cadence, and joint motion and increases metabolic cost during gait [3]. Some previous studies have evaluated the factors determining gait disturbance in patients with CNS demyelinating disease and reported that various factors, such as fatigue, balance problems, muscle mass and strength, and physical activity are associated with gait dysfunction [4–7]. Accurate and extensive knowledge of the factors associated with gait disturbance following the occurrence of CNS demyelinating diseases will enable clinicians and researchers to have a more accurate and well-balanced understanding of the disease. Moreover, the value of early detection of impairments in patients with CNS demyelinating diseases is highlighted by recent advances in treatment, particularly the availability of disease modifying drugs. The ability to identify gait and balance abnormalities in the early stages of CNS demyelinating diseases may provide a rationale for cohort selection targeting more aggressive therapeutic interventions. Therefore,in the current study, we investigated the association between gait function in patients with MS/NMO with mild disability and various clinical variables, such as disease duration, type of medication used, body mass index (BMI), muscle strength, cognitive deficit, depression, and fatigue.

## Methods

### Participant

Our study was approved by the Ethics Committee (IRB 2021–03-004) of Ulsan University Hospital and conducted in accordance with the principles of the Declaration of Helsinki for human experiments. All methods were carried out in accordance with relevant guidelines and regulations. Written informed consent was provided by all participants. We recruited consecutive patients with CNS demyelinating diseases between March 2021 and July 2022.

The inclusion criteria were as follows: diagnosis of MS or NMO according to the international consensus diagnostic criteria [8, 9], a follow-up period of more than 3 months from acute phase, and ability to walk independently (MS/NMO patients with Expanded Disability Status Scale (EDSS) ≤ 4.5). The patients who could not perform several self-questionnaire and tasks were excluded.

### Demographic data

The demographic or clinical information of patients, including age, sex, BMI, and date of symptom onset were recorded.

### Skeletal muscle index

Whole-body bioimpedance analysis (BIA) measurements were performed using an InBody S10 device (model JMW140; Biospace Co. Ltd., Seoul, Korea).

Skeletal muscle mass (SMM) was calculated using the Janssen equation [10]:

$$\mathrm{SMM}\hspace{0.17em}(\mathrm{kg})=\lbrack\mathrm{Ht}^2/\mathrm R50\times0.401)\hspace{0.17em}+\hspace{0.17em}(\mathrm{sex}\times3.825)\hspace{0.17em}+\hspace{0.17em}(\mathrm{age}\times-0.071)\rbrack+5.102$$

where Ht is height in centimeters, R50 is bioimpedance analysis resistance in ohms, for sex, men = 1 and women = 0, and age is in years.

The skeletal muscle index (SMI) was calculated using the following equation [11]:

$$S\mathrm{MI}\hspace{0.17em}(\mathrm{kg}/\mathrm m^2)=\mathrm{SMM}/\mathrm{Ht}^2$$

### Expanded disability status scale

The degree of disability was measured using the EDSS. The EDSS score at the last follow-up was measured by the same neurologist (SY Kim) [12].

### Gait analysis

A computer-based instrumented walkway system (Walk-way MG-1000, Anima, Japan) was used for gait analysis [13, 14]. This system measures the temporospatial parameters of gait by analyzing on/off signals between the patient’s foot and the surface of the sensors at a sampling frequency of 100 Hz [13, 14]. The length and width of the system’s walkway are 4.8 m and 0.82 m, respectively [13, 14]. During gait, data were obtained and processed automatically by the software embedded in the system.

### Experimental procedure for gait analysis

Participants wearing short pants were asked to get on a walkway and walk barefoot along a 12 m straight line, including 3.5 m in the front and 3.5 m beyond the end of walking path [13, 14]. Each participant performed one trial at a subjectively determined comfortable speed. A physical therapist with more than 20 years of experience performed gait analysis for all patients included in this study.

### Parameters of gait analysis

Measurements for temporal parameters, such as stance, swing, and double stance phase and stride were obtained in seconds. In addition, the duration of the stance, swing, and double stance phases during the total gait cycle for both lower limbs were expressed as percentages. Measurements of the spatial parameters, stride and step length and step width were obtained in centimeters. Additionally, gait speed (cm/s), gait angle (degrees), toe out angle (degrees), and cadence (steps) of each lower limb were measured. Stride length means distance from the first foot contact to another foot contact. It is the same meaning as the distance of one step. Step width means width between right and left with a heel contact to another heel contact. Gait angle means angle which is carried from the connected line of both heels between right and left foot against the forward direction. Toe out angle means open angle of a toe against the forward direction. Cadence means step per minute. For statistical analysis, the average values of both sides of all temporal and spatial parameters were used. All measurement values are presented as the mean ± standard deviation.

### Hand grip strength

Hand grip strength was measured using a digital grip strength dynamometer (TKK 5401 GRIP D; Takei, Japan), which measures between 5 and 100 kg of force [15]. The dynamometer was held by the testing hand in a neutral, comfortable position with 90° flexion of the index finger [15]. Participants performed three trials for the dominant hand and were instructed to squeeze the grip continuously with full force for at least 3 s [15]. The average of the three trials for each hand was recorded. For statistical analysis, the average grip strength of both hands was used.

### Instruments for assessing depression and cognition

Depression was evaluated using the Beck Depression Inventory score II (BDI), which is composed of 21 items with scores ranging from 0 to 63 and higher scores indicating more severe depression [16]. To screen patients who present with mild cognitive complaints, the Montreal Cognitive Assessment (MOCA) was used. The MoCA is a 30-point test that takes 10 min and includes short-term memory recall, visuospatial ability, executive function, attention-concentration-working memory, language, and orientation to time and place. In this study, the validated Korean version of the MOCA was used [17, 18].

### Functional assessment of chronic illness therapy-fatigue scale

The severity of fatigue was assessed using the Korean version of the Functional Assessment of Chronic Illness Therapy-fatigue scale (FACIT-fatigue), which is a 13-item questionnaire that assesses self-reported fatigue and difficulty in daily activities due to fatigue; its final score ranges from 0 to 52, with higher scores indicating less fatigue [19].

#### Statistical analysis

To identify the correlations between the gait-analysis clinical parameters, Pearson’s correlation test was used. Using only the parameters that were significant in the Pearson’s correlation test, multiple linear regression analysis was performed. To identify the variables affected by multicollinearity and the strength of the correlations, multicollinearity testing with variance inflation factors (VIFs) was performed. Multicollinearity was considered to be present when the VIF was higher than 5–10. Multiple linear regression tests with stepwise methods were performed after discarding parameters that showed multicollinearity. Statistical analyses were performed using the Statistical Package for the Social Sciences for Windows and the R package for Windows (version 2.15.2; R Foundation for Statistical Computing, Vienna, Austria).

## Results

### Patient characteristics

A total of 33 patients with CNS demyelinating disease (MS or NMO) of the CNS (5 men and 28 women, 17–68 years of age, 148.1–180.1 cm in height, and 43–108.7 kg in weight) were investigated in this study. Among the 33 patients with demyelinating disease of the CNS, 14 had MS, and the remaining had NMO. The results of the clinical assessments and gait analysis are presented in Table 1. There were no significant differences in clinical parameters between the MS and NMO patients (Table 1).

**Table 1:** Demographic and clinical characteristics of included patients

	MS	NMO	Total Patients	*P*-value
Age (years)	38.07 ± 13.76	50.68 ± 13.1	45.33 ± 14.62	0.12
Facit fatigue score	38.14 ± 11.34	33.89 ± 10.94	35.70 ± 11.14	0.20
Weight (kg)	63.39 ± 17.26	63.84 ± 15.80	63.65 ± 16.17	0.99
Height (cm)	159.10 ± 6.15	159.87 ± 7.63	159.54 ± 6.95	0.65
SMI muscle mass	8.53 ± 1.30	8.54 ± 1.42	8.54 ± 1.35	0.90
Muscle mass	21.74 ± 4.65	22.05 ± 5.49	21.92 ± 5.07	0.87
BMI (kg/m^2^)	24.96 ± 6.06	24.72 ± 5.18	24.82 ± 5.48	0.90
MOCA score	23.79 ± 7.31	26.58 ± 2.99	25.39 ± 5.36	0.38
EDSS score	1.93 ± 1.52	1.97 ± 1.14	1.96 ± 1.29	0.84
K-BDI score	16.71 ± 11.00	16.89 ± 11.95	16.82 ± 11.38	0.93
Disease duration (days)	2350.29 ± 1838.85	1837.05 ± 1468.59	2054.79 ± 1627.35	0.51
Gait analysis				
Stride length (cm)	90.32 ± 17.06	88.63 ± 15.02	89.35 ± 15.68	0.53
Step length (cm)	45.11 ± 8.60	44.36 ± 7.45	44.68 ± 7.84	0.48
Step width (cm)	11.46 ± 4.42	9.79 ± 3.07	10.50 ± 3.74	0.40
Gait angle (º)			14.18 ± 7.08	
Toe angle (º)	5.44 ± 5.65	7.57 ± 5.49	6.67 ± 5.57	0.29
Stride phase (sec)	45.34 ± 8.54	44.85 ± 7.78	45.06 ± 7.98	0.76
Swing phase	5.96 ± 2.06	5.03 ± 1.59	5.42 ± 1.83	0.44
Double phase	7.93 ± 4.92	6.72 ± 3.15	7.24 ± 3.98	0.60
Gait speed	75.60 ± 19.21	72.26 ± 17.51	73.68 ± 18.04	0.42
Cadence	99.73 ± 11.30	96.64 ± 10.95	97.95 ± 11.03	0.51
Hand grip power				
Non-dominant side			20.48 ± 9.16	
Dominant side			22.50 ± 9.26	
Average	20.52 ± 8.34	22.75 ± 9.63	21.80 ± 9.04	0.40
Functional score				
Motor	0.71 ± 0.99	0.47 ± 0.70	0.58 ± 0.83	0.63
Cerebellum	0.21 ± 0.80	0.11 ± 0.32	0.15 ± 0.57	0.90
Brainstem	0.14 ± 0.36	0.05 ± 0.23	0.09 ± 0.29	0.68
Bladder	0.36 ± 0.75	0.58 ± 0.77	0.48 ± 0.76	0.40
Visual	0.43 ± 0.65	0.21 ± 0.54	0.30 ± 0.59	0.36
Cerebral or mental	0.71 ± 0.91	0.26 ± 0.45	0.45 ± 0.71	0.19

*SMI* skeletal muscle index, *BMI* Body mass index, *MOCA* Montreal cognitive assessment, *EDSS* Expanded disability status scale, *K-BDI* Korean version of Beck's Depression Inventory

### Correlation between gait-analysis and clinical parameters

In the Pearson’s correlation test between gait analysis and clinical parameters, MOCA was found to be significantly correlated with gait speed, cadence, stride length, step length, and stride, stance, and double stance phase time (*p* < 0.05; Table 2). In the multivariate linear regression analysis, only gait speed had a significant positive correlation with MOCA (R^2^ = 0.452, *p* < 0.001, Table 3). In addition, EDSS scores were significantly correlated with gait speed, stride length, step length, stride phase time, swing phase time, and cadence (*p* < 0.05; Table 2). In the multivariate linear regression analysis, only the stance phase time was significantly negatively correlated with EDSS (R^2^ = 0.611, *p* < 0.001; Table 3).

**Table 2:** Correlation table for clinical parameters and gait-analysis parameters

Variable	MOCA	EDSS	FFS	SMI	MM	BMI	BDI	DD	HGP	SdL		SpL	SpW	TA	SdP	StP	SwP	DP	GS	CD
MOCA	1	-.563* (0.001)	.268 (0.131)	.122 (0.499)	.161 (0.370)	.167 (0.354)	-.148 (0.412)	-.292 (0.099)	.379* (0.030)	.486* (0.004)		.486* (0.004)	-.224 (0.209)	-.096 (0.595)	.492* (0.004)	.499* (0.003)	-.159 (0.376)	-.364* (0.038)	.500* (0.003)	.388* (0.026)
EDSS		1	-.191 (0.286)	-.291 (0.100)	-.377* (0.031)	-.177 (0.324)	.033 (0.854)	.358* (0.041)	-.216 (0.228)	-.595* (0.000)		-.579* (0.000)	.112 (0.535)	.254 (0.155)	-.608* (0.000)	-.642* (0.000)	.188 (0.295)	.464* (0.007)	-.616* (0.000)	-.509* (0.002)
FFS			1	-.050 (0.781)	.131 (0.466)	.010 (0.958)	-.695* (0.000)	.124 (0.491)	.314 (0.076)	.248 (0.164)		.251 (0.159)	.079 (0.061)	-.105 (0.562)	.192 (0.285)	.213 (0.234)	.097 (0.590)	-.049 (0.787)	.218 (0.224)	.225 (0.208)
SMI				1	.891* (0.000)	.810* (0.000)	.273 (0.124)	-.121 (0.503)	.227 (0.205)	.138 (0.442)		.131 (0.468)	.003 (0.985)	-.018 (0.921)	.105 (0.561)	.128 (0.477)	-.021 (0.907)	-.004 (0.983)	-.037 (0.836)	-.100 (0.579)
MM					1	.657* (0.000)	.111 (0.540)	-.120 (0.508)	.302 (0.088)	.256 (0.150)		.229 (0.199)	.095 (0.598)	-.230 (0.199)	.192 (0.285)	.237 (0.184)	.056 (0.756)	.004 (0.982)	.029 (0.873)	-.087 (0.629)
BMI						1	.188 (0.294)	.006 (0.973)	.110 (0.544)	.135 (0.454)		.149 0.409	-.072 (0.689)	.256 (0.151)	.102 (0.574)	.131 (0.466)	-.018 (0.921)	-.004 (0.983)	-.001 (0.996)	-.050 (0.782)
BDI							1	-.275 (0.121)	-.153 (0.396)	-.198 (0.269)		-.223 (0.211)	.003 (0.967)	.191 (0.287)	-.233 (0.192)	-.169 (0.347)	-.062 (0.733)	.096 (0.597)	-.169 (0.348)	-.040 (0.825)
DD								1	.139 (0.440)	-.292 (0.099)		-.269 (0.130)	.180 (0.315)	.202 (0.260)	-.228 (0.201)	-.345* (0.049)	.226 (0.206)	.404* (0.020)	-.349* (0.047)	-.273 (0.125)
HGP									1	.098 (0.588)		.084 (0.642)	.042 (0.817)	.148 (0.410)	.052 (0.775)	.041 (0.822)	.030 (0.868)	.029 (0.872)	.013 (0.942)	.132 (0.463)
SdL										1		.980* (0.000)	-.186 (0.301)	-.274 (0.123)	.955* (0.000)	.978* (0.000)	-.162 (0.368)	-.528* (0.002)	.907* (0.000)	.551* (0.001)
SpL SpW												1	-.172 (0.337) 1	-.274 (0.123) -.026 (0.884)	.960* (0.000) -.250 (0.160)	.972* (0.000) -.199 (0.267)	-.145 (0.421) .952* (0.000)	-.521* (0.002) .832* (0.000)	.907* (0.000) -.228 (0.202)	.524* (0.002) -.153 (0.396)
TA														1	-.221 (0.217)	-.279 (0.116)	.047 (0.796)	.129 (0.474)	-.302 (0.087)	-.135 (0.453)
SdP															1	.944* (0.000)	-.208 (0.246)	-.574* (0.000)	.868* (0.000)	.491* (0.004)
StP																1	-.190 (0.289)	-.569* (0.001)	.912* (0.000)	.559* (0.001)
SwP																	1	.861* (0.000)	-.204 (0.254)	-.148 (0.410)
DP																		1	-.580* (0.000)	-.417* (0.016)
GS																			1	.799* (0.000)
CD																				1

Correlation coefficient (*p*-value). *MOCA* Montreal cognitive assessment, *EDSS* Expanded disability status scale, *FFS* Facit fatigue score, *SMI* skeletal muscle index, *MM* Muscle mass, *BMI* Body mass index, *BDI* Beck's Depression Inventory, *DD* Disease duration, *HGP* Handgrip power, *SdL* Stride length, *SpL* Step length, *SpW* Step width, *TA* Toe angle, *SdP* stride phase, *StP* Stance phase, *SwP* Swing phase, *DP* Double phase, *GS* Gait speed, *CD* Cadence *p-value < 0.05

**Table 3:** Multiple linear regression analysis among various clinical parameters in patients with MS/NMO

Dependent variable	Independent variables	R^2^	Beta coefficient	Standard error	Odd ratio (95% CI)	*P* value
MOCA	Gait speed	0.452	0.673	0.179	0.541 ~ 1.271	< 0.001
EDSS	Stance phase time	0.611	-0.238	0.034	-0.308 ~ -0.168	< 0.001
Hand Grip Strength	Muscle Mass at BIA	0.649	2.227	0.584	1.033 ~ 3.421	0.001
FACIT Fatigue Scale	K-BDI score	0.529	-0.712	0.121	-0.958 ~ -0.466	< 0.001

*MS* Multiple sclerosis, *NMO* Neuromyelitis optica, *MOCA* Montreal cognitive assessment, *EDSS* Expanded disability status scale, *BIA* Bioimpedance analysis

### Other correlations

In the Pearson’s correlation test, the FACIT-fatigue score was significantly correlated with the results of BDI (*p* < 0.05). In the multivariate linear regression analysis, the FACIT- fatigue score also had a significantly negative correlation with the results of BDI (*p* < 0.001; Table 3). In addition, hand grip power was significantly correlated with BMI, SMI, and SMM, as assessed by BIA (*p* < 0.05). However, in the multivariate linear regression analysis, only SMM, had a significant positive correlation with hand grip strength (R^2^ = 0.649, *p* = 0.001; Table 3).

## Discussion

Gait speed showed a significantly positive correlation with the results of cognitive function (MOCA), and stance phase time showed a significantly negative correlation with the degree of disability (EDSS) in patients with MS/NMO with mild disability. In other words, the better the patients’ cognitive function, the faster their gait speed. In addition, the higher the patient’s disability, the shorter is the stance phase time.

The positive correlation between cognitive function and gait speed may be because both cognition and gait function are affected by the degree of brain involvement. As brain involvement by MS/NMO disease increases, gait speed slows down and cognition decreases [20]. Another possible reason for the correlation between cognitive function and gait speed may be the presence of postural compensatory strategies by cognitive functions in humans. Accumulating data have suggested that cognitive and postural tasks may interfere with each other when performed concomitantly. Therefore, it has been hypothesized that a like exists between the cognitive domain and postural control, defined as cognitive-posture interference/cognitive-motor interference [21–23]. Therefore, in patients with MS/NMO disease those with high cognitive function are believed to better complement the relatively deteriorated compensatory strategy.

In addition, the results of this study showed a negative correlation between the degree of disability and the stance phase time. The core function of maintaining stability during the stance phase is the ability to stably support the body weight on one leg. Patients with severe disability have difficulty sustaining stability on one leg, which lengthens the stance phase time. The weakness of hip extensor muscles and ankle plantar flexor power, and restricted ankle plantar flexion during the toe off during stance phase, which have been reported in the previous studies, also may lengthen the stance phase time in patients with MS/NMO [24–26].

In addition, the FACIT-fatigue score had a significant negative correlation with the K-BDI score, which indicates that patients with more fatigue tend to be more depressed. This result is consistent with that of previous studies [27–29]. In previous studies, the authors found that patients with fatigue had more severe depression than those without fatigue, and that fatigue score correlated with depression scores. The authors of these studies believed that fatigue has a significant correlation with depression because fatigue in patients with NMO/MS could be secondary to mood disorders, such as depression.

In addition, hand grip strength was significantly correlated with SMM, as assessed by BIA in this study. This is believed to be because the greater the muscle mass, the more muscle power it is likely to have, and handgrip power is a representative parameter to measure it. This is consistent with the results of a previous study that showed a positive correlation between hand grip strength and SMM assessed by BIA in patients with sarcopenia and multiple sclerosis [30].

Although very few studies have investigated the correlations between gait analysis and clinical data in patients with NMO, some studies have investigated the correlations between gait analysis and various clinical parameters in patients with MS. D'Orio et al. [31] reported that lower processing speed and intelligence quotient predicted slower gait speed in patients with MS and poorer verbal memory predicted an increased frequency of falls. Kalron et al. [32] reported a significant reverse correlation between the degree of gait impairment and cognitive motor skills assessed using a computerized cognitive battery test. Hsieh et al. [33] also reported that a slower cognitive processing speed (assessed by symbol digit modalities) was significantly associated with greater gait instability. The correlations between cognition and gait impairment in previous studies are consistent with the results of our study. Considering the close correlation between cognitive and gait function, training for improving cognitive function in patients with CNS demyelinating disorders might be helpful in improving gait function by repairing deteriorated compensatory strategies. Moreover, these results may imply that early detection of a decrease in gait speed and an increase in stance phase time can predict the progression of cognitive impairment in patients with MS/NMO with mild disability.

This study has a few limitations. First, a small number of patients with MS and NMO were enrolled. Therefore, it was difficult to determine the correlation between each group of patients with MS and NMO. Studying the correlation of gait disturbance in each disease with more patients in the future will be more meaningful. Second, no data were collected in this study that could affect outcomes, such as the presence of concomitant diseases, medications the patient is taking, or whether the patient has undergone rehabilitation treatment. Further studies enrolling more patients will be necessary in the future. Lastly, since we enrolled only patients with CNS demyelinating disorder and mild disability who could walk, further studies are required to examine trends in the overall patient population.

## Conclusion

In our patients with MS/NMO, cognitive impairment was significantly correlated with gait speed, and the degree of disability was significantly correlated with stance phase time. Our findings suggest that gait disturbance and cognitive deficits in patients with MS/NMO are closely correlated. These results may imply that early detection of a decrease in gait speed and an increase in stance phase time can predict the progression of cognitive impairment in patients with MS/NMO with mild disability. In other words, this may also mean that caring for cognitive function in patients with MS/NMO might be helpful in maintaining good walking ability. In addition, we believe that rehabilitative training in patients with severe disability should focus on improving stability during the stance phase of the gait cycle. However, further studies with a larger number of patients with MS/NMO are warranted for better evaluation.

## Data Availability

All original data can be obtained via email correspondence to bdome@uuh.ulsan.kr, and all charts in this study are presented in the article.
